# Cross-group friendship and collective action in community solidarity initiatives with displaced people and resident/nationals

**DOI:** 10.3389/fpsyg.2023.1042577

**Published:** 2023-04-03

**Authors:** Megan Vine, Ronni Michelle Greenwood

**Affiliations:** Department of Psychology, University of Limerick, Limerick, Ireland

**Keywords:** cross-group friendship, intergroup contact, political solidarity, collective action, displaced people, social cohesion

## Abstract

**Introduction:** In Ireland, people seeking asylum (displaced people) receive accommodation in a system called “Direct Provision” (DP) while they wait for their applications for protection to be processed. The living conditions of DP have been described as illegal and inhumane by national and international human rights groups, and the system exacerbates the social exclusion of displaced people. Community responses to DP by displaced people and resident/nationals of Ireland include the creation of informal groups called community solidarity initiatives (CSI), through which cross-group friendships are forged by participation in shared cultural activities. We hypothesized that, compared to non-CSI participants, participants of CSI would report more cross-group friendships, and that more cross-group friendships would predict stronger collective action intentions to support the campaign to end DP, especially among resident/nationals.

**Methods:** We recruited residents/nationals and displaced persons with and without CSI experience to complete a self-report questionnaire (*n* = 199), measuring cross-group friendship, collective action intentions, and intergroup attitudes. Data were collected between July 2020 and March 2021, using a combination of online and paper surveys. We conducted ANOVA and conditional process analyses on the data to test our hypotheses.

**Results:** As predicted, CSI participants reported more contact with cross-group friends and stronger collective action intentions than non-participators. Conditional process analysis indicated that CSI participation facilitated resident/nationals’ political solidarity with displaced people through cross-group friendship.

**Discussion:** Findings identify the role of group membership in the relationship between contact and collective action for migrant justice, illustrating the potential of CSI to bolster intergroup solidarity and social cohesion through shared activities and cross-group friendship. As such, findings make an important contribution to the literature on intergroup contact, solidarity, and social cohesion, and will be relevant for community practitioners, civil society organisations, NGOs, and policy makers.

## Introduction

In Ireland, people seeking asylum (hereafter displaced people^1^) are housed in congregate settings through the Direct Provision (DP) system while their applications for international protection are processed. This carceral system segregates and excludes displaced people from the wider Irish community through disempowering regulations and restrictions on their rights, with multiple negative effects (Loyal and Quilley, 2016; DORAS, 2020). Community solidarity initiatives (CSI) challenge this *status quo* by bringing displaced people and residents/nationals together around shared activities, facilitating resistance to negative social representations and recognition of valued identities (Vine and Greenwood, 2020, 2022). CSI are an emerging context of contact among displaced people and residents/nationals, and the correlates of participation for resident/nationals and displaced people are not fully understood. We investigated whether and how participation in CSI is related to cross-group friendships, collective action intentions, and intergroup attitudes, for both resident/nationals and displaced people. We also examined whether the relationship between CSI participation and collective action intentions was mediated by cross-group friendship, and whether the pattern of association was different for residents/nationals than for displaced persons. In the following sections we provide an overview of the research context, followed by a review of the relevant literature on contact and collective action that provided the basis for our hypotheses.

## Research context

Direct Provision is a system of congregated accommodation for international protection applicants in Ireland. DP centers are privately-run hostels, hotels, and B&Bs, often in isolated and rural locations that lack appropriate services and supports. People living in DP report delays and dysfunction in the immigration system, protracted stays in cramped accommodation, a lack of privacy, lack of access to cooking facilities, and disempowering regulations on their autonomy (Breen, 2008; Stapleton, 2012; Loyal and Quilley, 2016; DORAS, 2020; Asylum Information Database, 2022). The COVID-19 pandemic worsened these conditions for people in DP, many of whom were cut off from support services and were unable to physically distance from non-family members in congregated settings, which had a disastrous impact on displaced people’s physical and mental health during the pandemic (DORAS, 2020; Irish Refugee Council, 2020). People seeking asylum do not qualify for state benefits; however, they receive free medical care and a weekly stipend of €38.80 per adult. As non-citizen residents, displaced people may vote or stand in local elections, but are barred from other formal means of political participation. A 12-month permit to access the labor market is possible for people who have not yet received a decision on their application for international protection after 6 months in Ireland. Displaced people who have not yet gained refugee status may access free education up to Leaving Certificate (NFQ level 5), however, they face structural and financial barriers to accessing university education (Asylum Information Database, 2022).

People seeking asylum in Ireland arrive from a wide range of countries, but the most common are Zimbabwe, Nigeria, Somalia, Georgian, Algeria, and Afghanistan (Department of Justice, 2022). Although Ireland is becoming increasingly ethnically diverse, Irish society is still overwhelmingly White, Catholic, and characterized by structural and interpersonal racism (Joseph, 2017). Reports on racism consistently identify people from Black backgrounds as the primary targets of discrimination in Ireland (McGinnity et al., 2018a,b, 2020), and many people living in DP are doubly stigmatized in Irish society by their asylum-seeking status and their racial categorization (Lentin, 2022). Since the invasion of Ukraine in March 2022, refugee NGOs and activists have criticized the double standards in Ireland’s treatment of displaced people from White and non-White backgrounds. Ireland has accepted (73,490) Ukrainian refugees since March 2022 (BBC News, 2023); however, Ukrainian people qualify for Temporary Protection within the EU, which affords them fast-tracked access to Irish state supports and services. Furthermore, there are additional supports for Ukrainian people to access accommodation, so although many are living temporarily in congregated settings, they are separate from the DP system. In 2021, the Irish government pledged to abolish DP within 5 years, and replace it with a more “humane, person-centered” system (Department of Children, Equality, Disability, Integration and Youth, 2021); however, in the context of housing scarcity and the recent increases of people with protection needs, they are unlikely to achieve this goal.

Although far-right, anti-immigration groups do not have a strong foothold in Ireland, in contrast to other EU nations such as Hungary and Italy, there are small numbers of far-right groups and individuals who have become increasingly visible and vocal in their opposition to migrants, asylum seekers, and refugees in Ireland (Kelleher, 2022). During and after the Syrian refugee reception crisis, increased media coverage of discrimination against displaced people in Ireland corresponded with an upswell in public solidarity with people in DP, when 832 people pledged to host Syrian refugees in their homes (Irish Red Cross, 2022). Community solidarity initiatives (CSI) emerged from this context of increased focus on issues affecting displaced people, and they were established by displaced people and their allies around Ireland as a response to the social exclusion and segregation caused by DP. CSI are informal groups that range from running clubs and support networks to food sharing and cultural celebrations. Despite the diversity in approaches, all CSI are founded on a shared value: that everyone benefits when displaced people are fully included in Irish society. These initiatives build informal networks of support between residents/nationals and displaced people through participation in shared activities. The most visible of these groups is the nationwide Sanctuary Runners network of running clubs, who have the motto “Solidarity, Friendship and Respect” (Sanctuary Runners, 2022). Other groups like The Melting Pot Luck in Galway organize community events to bring displaced people and resident/nationals together in a welcoming environment. Some CSI-type initiatives are more explicitly political, for example Movement of Asylum Seekers in Ireland (MASI) and Refugee and Migrant Solidarity Ireland (RAMSI), who organize solidarity events that directly support the campaign to end DP.

### Social cohesion, solidarity, and CSI

According to Schiefer and van der Noll (2016), “social relations, sense of belonging and orientation toward the common good” are key components of social cohesion, which all intersect with the broader concept of “solidarity” (p. 581). Research on social cohesion in diverse contexts tends to focus on aspects of social relations such as trust, inter-group attitudes, and shared identity (van der Meer and Tolsma, 2014; Abascal and Baldassarri, 2015; Demireva, 2019; Reimer et al., 2021). Some researchers have posited that social cohesion and diversity are at odds with one another. For example, Putnam (2007) described lower levels of intergroup trust in ethnically diverse neighborhoods in the United States (e.g., Putnam, 2007). In contrast, researchers in United Kingdom and EU contexts (van der Meer and Tolsma, 2014; Demireva, 2019) reported that intergroup trust in diverse neighborhoods was lower only for White participants (Abascal and Baldassarri, 2015). Furthermore, where intergroup attitudes are positive, diversity and social cohesion are more likely to coexist (Laurence et al., 2018).

Intergroup solidarity involves people from different backgrounds working together toward the same cause (Subašić et al., 2008; Neufeld et al., 2019) and as such, is a key component of social cohesion in a diverse society (Schiefer and van der Noll, 2016). Structural racism influences immigration policies that shape the conditions displaced people face in their receiving countries, including where they can live (Benson, 2022; Lentin, 2022). When displaced people are forced to live in congregated settings like DP, they are not able to fully participate in community activities, which weakens social cohesion. Through CSI, displaced people and resident/nationals create opportunities for participation in inclusive community activities, facilitating cross-group friendships. In the present study, we investigated the potential for CSI to facilitate solidarity in a diverse and unequal context through participation in inclusive cultural activities.

### Contact with cross-group friends and collective action

Classical contact research focuses on the *contact effect*, the finding that sustained, positive intergroup contact reduces intergroup prejudice, especially advantaged groups members’ prejudice toward disadvantaged groups (Allport, 1954; Pettigrew and Tropp, 2006). However, effect sizes are often small (Paluck and Green, 2019), and power asymmetries produce different experiences of intergroup contact for members of advantaged and disadvantaged groups (Hopkins et al., 2007). Furthermore, in contexts of entrenched intergroup conflict and inequality, people from different ethnic, social, or religious groups often *informally segregate* (Maoz, 2011; McKeown and Dixon, 2017; Dixon et al., 2020). Group status also influences preferences for intergroup contact, such that advantaged group members prefer not to discuss differences in group status and power (Saguy and Kteily, 2014), while disadvantaged group members prefer to share their experiences of inequality (Bruneau and Saxe, 2012). For these and other reasons, advantaged and disadvantaged group members are more likely to seek out contact when encounters or interventions address *both* groups’ individual and group-level needs, when individual and group level differences are acknowledged, and when shared identities can be formed (Kauff et al., 2020).

Collective action research has traditionally focused on the predictors of collective action engagement for disadvantaged group members on behalf of their own group, such as strength of ingroup identification, perceptions of injustice, and perceived efficacy of ingroup actions (van Zomeren et al., 2008; Dixon et al., 2016; van Zomeren, 2019; Dixon and McKeown, 2021). The Social Identity Model of Collective Action (SIMCA) identifies these as core predictors of collective action, so that if a member of a disadvantaged group identifies strongly with their ingroup, perceives intergroup inequality to be illegitimate, is angry about it, and believes their group can be effective in remedying the injustice, then they are more likely to engage in collective action in support of their ingroup (van Zomeren et al., 2008; Hässler et al., 2020a). Recent research has identified some factors that predict advantaged group members’ collective action in support of disadvantaged groups’ rights, such as intimate intergroup contact and inclusive identification (Reimer et al., 2017; Radke et al., 2020; Hässler et al., 2020a). Intergroup contact may have a complex relationship with advantaged and disadvantaged group members’ collective action intentions, however (Reimer et al., 2017; Kotzur et al., 2019; Hässler et al., 2022).

For advantaged group members, positive contact and cross-group friendships predict stronger collective action intentions (Reimer et al., 2017; Hässler et al., 2020a; Tropp et al., 2021). Content of contact seems to matter; when friendships involve communication about differences in group power, advantaged friends are more willing to take collective action in solidarity with their disadvantaged friend’s cause (Tropp et al., 2021). In contrast, for disadvantaged group members, positive intergroup contact predicts weaker intentions to participate in collective action on behalf of their group (e.g., Reimer et al., 2017; Hässler et al., 2020a), perhaps because positive contact is associated with weaker perceptions of injustice, lower support for social change policies, and intentions to take collective action to address injustice experienced by members of their group (van Zomeren et al., 2008; Saguy et al., 2009; Dixon et al., 2010, 2012). Certain kinds of communications in cross-group friendships may bolster collective action intentions of members of disadvantaged groups, such as when advantaged group members recognize and criticize intergroup inequality as illegitimate (Becker et al., 2013; MacInnis and Page-Gould, 2015; MacInnis and Hodson, 2019; Hässler et al., 2020a). Thus, opportunities for individuals to develop cross-group friendship *and* discuss their similarities and differences may indirectly strengthen collective action intentions for members of both groups (MacInnis and Page-Gould, 2015; MacInnis and Hodson, 2019). We believe CSI provide such opportunities for their members.

Cross-group friendships have long been considered an *ideal* form of intergroup contact, because they are likely to meet the “optimal conditions” of cooperation, common goals, and equal-status interactions (Pettigrew, 1998; Davies et al., 2011). Accordingly, they are more effective in reducing intergroup prejudice than traditional contact interventions (Pettigrew and Tropp, 2006). Close and engaged cross-group friendships have the strongest effect on intergroup attitudes (Wright et al., 1997; Davies et al., 2011). Through such relationships, positive attitudes toward the cross-group friend may be generalized to the outgroup through processes of *categorization* (Brown et al., 1999), while at the same time individuating outgroup members through *decategorization* (Brewer and Miller, 1984) and *recategorization* under a superordinate identity (Gaertner et al., 1999). There are important limitations to this effect, that are relevant here. For example, in a context of entrenched inequality, the effect of a contact intervention was limited to the situation (Mousa, 2020), and a large-scale experiment investigating the effects of a contact intervention with Youth found no effect on intergroup attitudes (Reimer et al., 2021). Accordingly, we are somewhat agnostic about the effect of CSI participation on intergroup attitudes, given the real power asymmetries that exist between displaced people and resident/nationals in this context. Nonetheless, we expect that given the likelihood of cross-group friendship in CSI, there will be a small effect on intergroup attitudes, at least for advantaged group members.

Collective action by displaced people, for displaced people is crucial in the campaign to end the Direct Provision system in Ireland, and political solidarity among resident/nationals and displaced people can help to sustain this effort. In Ireland, non-citizens’ voting rights are limited to local elections, and displaced people may also experience barriers to collective action related to experiences of oppression in their country of origin, or they may experience intersecting disadvantages related to their gender, religion, ethnicity, or other identity categories that block their political participation (Bekaj and Antara, 2018). People living in DP face additional barriers to involvement in protests, because doing so puts them at risk of harassment, threats, or even eviction by the management of their DP center (O’Toole, 2019). Despite the multiple barriers displaced people face to taking collective action, displaced people are not passive “victims,” on the contrary—many are active participants and leaders within their communities. Displaced people often engage in informal and relational forms of collective action such as mutual aid to support one another (Betts et al., 2018), and Movement of Asylum Seekers in Ireland (MASI) have demonstrated how an asylum seeker-led movement can mobilize strong support across society to change State policies (Lentin, 2014). Indeed, refugee and migrant-led organizations like MASI are important sources of collective action and mutual-aid by displaced people that deserve more recognition and support (Betts et al., 2018).

Community solidarity initiatives encompass a continuum of overtly political and non-political groups; however, most CSI emphasize human connection above political motivations for participation. Resident/nationals’ *apolitical* solidarity with refugees has been criticized for reinforcing problematic power dynamics, positioning refugees as “vulnerable” beneficiaries and resident/nationals as benevolent allies. Nevertheless, there is also potential for “spaces of encounter” in solidarity groups to build intergroup bonds that subvert power asymmetries and sustain action for social change (Fleischmann and Steinhilper, 2017; Ambrosini, 2022).

Qualitative research on resident/nationals’ and displaced people’s experiences of CSI suggests that CSI facilitate recognition of shared identities and affirmation of valued ingroup identities, which are important for individual and group needs to be met (Vine and Greenwood 2020, 2022; Kauff et al., 2020). For example, participants believed that friendships formed through CSI participation shifted attitudes and broadened acceptance of displaced persons in the wider community (Vine and Greenwood, 2022). Supportive bonds developed between resident/nationals and displaced people in CSI can be understood as a form of *relational solidarity*, which is important in laying the foundation for more political forms of solidarity (Subašić et al., 2008; Straehle, 2020). Although most CSIs are not *overtly* political in nature, they all aim to challenge the social exclusion caused and sustained by the DP system, meaning that CSI are at least *implicitly* critical of DP.

Collaborative intergroup contact affords advantaged group members opportunities to learn about the circumstances of less advantaged groups (Tropp et al., 2021), and resident/nationals have reported becoming more aware of displaced people’s realities through CSI (Vine and Greenwood, 2020). Accordingly, it is possible that due to this newfound awareness, resident/national participants of CSI discuss the illegitimacy and unfairness of this system with cross-group friends, which may fuel members of both groups’ commitment to collective action for displaced people’s rights (MacInnis and Hodson, 2019). Therefore, we expect that participants of CSI who have more contact with cross-group friends will demonstrate stronger collective action intentions. Our previous research and the contact literature emphasizes the key role of group status in influencing experiences and outcomes of contact (Vine and Greenwood 2020, 2022; Hässler et al., 2020a; Tropp et al., 2021). Thus, we expect that group membership (resident/national or displaced) will be a significant moderator of this relationship.

### The present study

**Figure 1:**
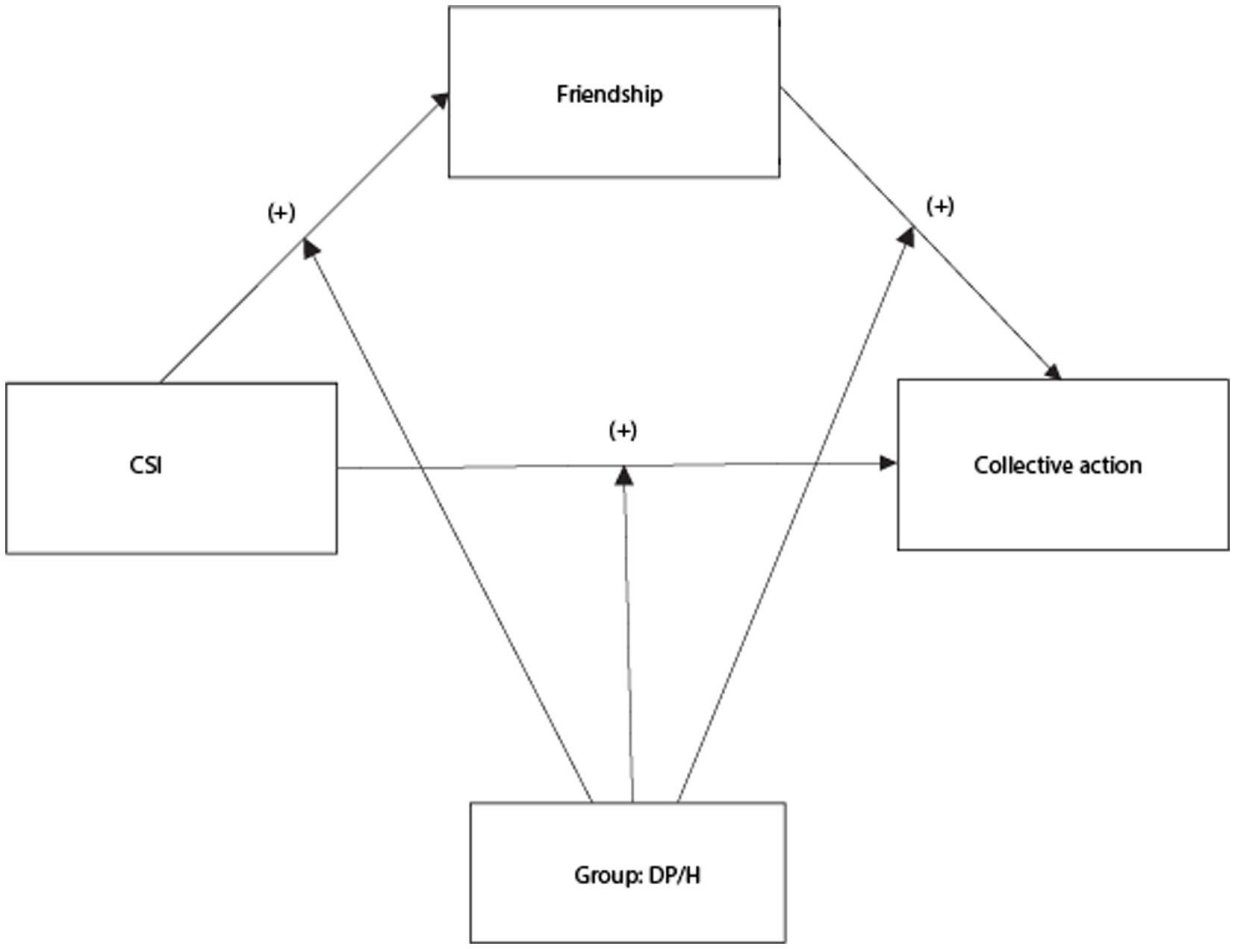
Hypothetical moderated mediation model.

We conducted a quasi-experimental investigation of the effects of CSI participation on resident/nationals’ and displaced persons’ cross-group friendships, collective action intentions, and intergroup attitudes. We hypothesized that residents/nationals and displaced people who participate in CSIs would have: (1) more contact with cross-group friends, (2) stronger collective action intentions in support of displaced people’s rights, and (3) more positive intergroup attitudes than those who do not participate in CSIs, with stronger effects for resident/nationals than displaced people. Furthermore, we hypothesized that: (4) cross-group friendship would mediate the relationship between CSI participation and collective action intentions, and that each path of this mediation this would be moderated by group membership, such that the positive effect would be stronger for resident/nationals than displaced people (see Figure 1).

## Materials and methods

This research forms part of a larger project in which we compared psychological and behavioral outcomes of participation in CSI for displaced and resident/national participants. Please see the Supplementary material for the complete questionnaire. We asked participants to indicate whether they had participated in CSI in the past year (Yes/No), and whether they were currently seeking asylum or had done so in the past 5 years (Yes/No), and created four groups based on their responses: (1) HCSI: resident/nationals who have participated in CSI, (2) HnCSI: resident/nationals who have not participated in CSI, (3) DPCSI: displaced people who have participated in CSI, and (4) DPnCSI: displaced people who have not participated in CSI.

### Participants

We conducted an *a priori* power analysis using G*Power3 for ANOVA with four groups, a medium effect size, and an alpha of 0.05, and found that a minimum sample of 416 was needed to achieve power of 0.8 (Faul et al., 2007). We received 265 questionnaires, of which 199 were retained for analysis. Of the 66 participants removed from the dataset, 56 had pervasive missing data, and nine were involved in overtly political CSI. One was removed after outlier analysis.

Participants’ demographic characteristics are described in Table 1. More than a third were Irish (37%). Other nationalities represented in our sample included: Zimbabwean (10%), Nigerian (9%), South African (6%) Malawian (3%), and Pakistani (3%). A third (33%) were living in County Galway at the time of data collection, 18% lived in Co. Mayo, 6% in County Dublin, 5% in County Cork, and 5% in County Limerick. A diversity of CSI and CSI activities were reported by participants who had engaged with CSI (Tables 2, 3).

**Table 1:** Sample demographic characteristics.

Demographic category	Subcategory	*n*	%
Condition	DPCSI	81	38.8
	DPnCSI	30	14.4
	HnCSI	52	24.9
	HCSI	46	22.0
	Total	209	100
Gender	Male	51	24.4
	Female	126	60.3
	Other	1	0.5
	Total	178	85.2
	18–26	14	6.7
Age	27–38	83	39.7
	40+	49	23.4
	50+	14	6.7
	60+	14	6.7
	70+	5	2.4
	Total	179	85.6
	Irish	61	29.2
Ethnicity*	Other White background	21	10.0
	African	72	34.4
	Other Black background	1	0.5
	Other Asian background	17	8.1
	Other (including mixed background)	7	3.3
	Total	179	85.6
	Irish citizen	64	30.6
Immigration status	EU citizen	13	6.2
	Asylum seeker	83	39.7
	refugee	9	4.3
	Stamp 4	7	3.3
	Stamp 2	1	0.5
	Stamp 1	1	0.5
	Total	178	85.2

* Ethnicity categories taken from Irish Census.

**Table 2:** Community solidarity initiatives.

CSI name^*^	*n*	%
Melting Pot Luck	20	9.6
Sanctuary Runners	7	3.3
English classes	2	1.0
Ballyhaunis Inclusion Project	10	4.8
Multicultural choir	2	1.0
Various initiatives	6	2.9
Health Hub Sanctuary Project	2	1.0
Refugee solidarity group	4	1.9
Community cooking	2	1.0
Something from There	2	1.0
Art classes	2	1.0
Mayo Intercultural Action	5	2.4
Cultural celebration	2	1.0
COPE	2	1.0
Macroom Food Festival	5	2.4
Cricket	2	1.0
Tidy Towns	2	1.0
Other^**^	22	11.0

* Free-entry responses collated by research team.

** All CSI representing less than 1% of the sample were combined into this category.

**Table 3:** CSI activities.

Activity^*^	*n*	%
Cooking/Food	54	25.8
Sport	28	13.4
Art & Craft	34	16.3
Music	31	14.8
Dance	22	10.5
Fashion/Textiles	19	9.1
Other	32	15.3

* Participants could select more than one type of activity per CSI.

There were important differences between the displaced and resident/national conditions of our study in terms of ethnicity, immigration status and nationality: people who identified as Black/African represented 69% of DPCSI and 43% of DPnCSI respectively, while people who identified as White/Irish represented 61.5% of HnCSI and 64.3% of HCSI, respectively. People mostly identified themselves as “asylum seekers” in DPCSI and DPnCSI (83 and 60% respectively), while people mostly identified themselves as “Irish citizens” in HCSI and HnCSI (69 and 64% respectively). Nigeria (16%), Zimbabwe (20%), and South Africa (11%) were most prevalent nationalities in DPCSI, and a similar pattern was observed in DPnCSI (Zimbabwe; 16.7%, Nigeria; 13.3%, Malawi; 13.3%, and Algeria; 11%). Irish was by far the most prevalent nationality in HCSI (85%) and HnCSI (69%), respectively. There were also some differences in gender and age across the groups. Women were over-represented across three conditions: DPCSI: 59%, HCSI: 81%, and HnCSI: 64%; however, there was an equal proportion of males and females (36.7%) who answered the gender question in the DPnCSI condition. Most participants’ ages fell within the 27–38 age category: DPCSI (61%), DPnCSI (40%), and HnCSI (23%); however, the HCSI condition contained a higher proportion of participants in the 40+ category (38%).

### Data collection

We recruited participants *via* online and paper surveys to ensure a broad reach, and to access participants living in Direct Provision centers. These methods were chosen to best suit the different cohorts we sought to recruit for our study, and we used different data collection strategies for both, which we explain below.

#### Online data collection

We used the online survey platform Qualtrics to collect most of the responses. We distributed a link to the survey to refugee and migrant community groups, NGOs, and civil society organizations to reach all four of our targeted subgroups. We also shared the survey more broadly within our University network, and among other community-based organizations to access resident/nationals who have not participated in CSI. Finally, the lead author engaged with individuals living in DP to serve as research assistants who could distribute the survey link among harder-to-reach displaced people who had not participated in CSI. The survey questions were worded to match the participants’ group memberships (HCSI, HnCSI, DPCSI, or DPnCSI). Online data collection took place from July 2020 to March 2021. We received a total of 132 valid responses to the online survey, representing 66% of the overall sample. Respondents to the online survey reflected all four groups under study, however DPnCSI and DPCSI were underrepresented, so we decided to do supplementary data collection in DP centers using paper surveys.

#### Paper data collection

To obtain additional response from the DPCSI and DPnCSI cohorts, we distributed (*n* = 200) paper questionnaires to displaced people living in four DP centers in different regions of Ireland. We selected this method based on feedback from people living in DP that residents were more likely to complete a paper questionnaire than an online questionnaire. The paper questionnaires included identical questions to the online survey. Four RAs living in DP centers distributed the paper versions to residents, and once the questionnaires were completed, the RAs collected them in sealed envelopes and returned them by mail to the first author. We received an additional 62 questionnaires (31% of the total sample) using this method.

Many residents in DP centers read and speak Arabic, so we also had the survey translated into standard Arabic to broaden our reach to potential participants who have low levels of English literacy. We used back-translation with two translators to ensure the translated surveys remained as close as possible to the meanings of the original (Wild et al., 2005). We distributed paper versions of the Arabic language survey (*n* = 20) in two Direct Provision centers; however, only a small number of surveys (*n* = 5) were completed in Arabic. Thus, we used several strategies to recruit a broad range of displaced people to participate in the study.

#### Analytic strategy

We conducted between-subjects univariate ANOVAs to compare outcomes in cross-group friendship, collective action and outgroup attitudes between resident/national and displaced participants and non-participants of CSI. Then, we conducted conditional process analysis, using moderated mediation model 59 of Hayes’ *PROCESS* macro (Hayes, 2012), with “CSI participation” as predictor, “cross-group friendship” as mediator, “collective action” as outcome, and “group” (resident/national or displaced) as moderator of all paths in the model.

### Measures

#### Cross-group friendship

We measured cross-group friendship with a single item derived from the *Contact Quantity Contact Quality* (CQCQ) scale (Islam and Hewstone, 1993), which captured the *amount of contact with outgroup friends* on a seven-point Likert scale from “never” to “daily.” The instructions for this item were adapted for each of the groups under study, instructing resident/national participants to think about contact with displaced friends and vice versa for displaced participants.

#### Collective action

Intentions to participate in collective action on behalf of displaced people were measured with an adapted version of a scale developed by Reimer et al. (2017). Seven items measured intentions to participate in different forms of collective action including: supporting political candidates who advocate for displaced people’s rights, participation in protests, signing a petition, joining a group of activists, attending events, defending displaced people’s rights to others, and supporting a displaced person who is facing discrimination. Participants were asked to rate how often they intended to engage in these actions in future on a six-point scale from “Never” to “Very often.” Internal consistency reliability was high for this scale (α = 0.89).

#### Outgroup attitudes

Outgroup attitudes were measured with an outgroup feelings thermometer (Converse et al., 1980), on which participants rated their warmth toward a specific outgroup on a scale from 0 to 100 degrees. All resident/national participants were asked to rate their feelings toward displaced people in Ireland, and all displaced participants were asked to rate their feelings toward the wider Irish community.

#### Demographics and participant characteristics

We asked all participants to report their age, gender, ethnicity, immigration status, location in Ireland and nationality. Categories for these were taken from the corresponding sections of the Irish Census form; however, we recognize that these categories are problematic because they conflate nationality with ethnicity in ways that do not reflect the lived reality of Black and mixed heritage Irish people (Ogoro et al., 2022). CSI participants were also asked to name their CSI, the type of activities they engaged with, and the level and frequency of their participation.

## Results

### Preliminary analyses

First, we assessed each variable for normality. Both skew and kurtosis variable were < 1.0 for all variables, indicating that they met the assumption of univariate normality (Weston and Gore, 2006). Chi-squared tests identified a significant association between our independent variables (CSI participation and group; *X*^2^_1,197_ = 0.27, *p* = <0.001). Next, we carried out independent samples *t*-tests to assess the relationships between our independent variables and our three dependent variables (cross-group friendship, collective action intentions and intergroup attitudes). There was a significant difference between CSI participants and non-participants (0 = CSI non-participant, 1 = CSI participant) in cross-group friendship (*t*_169_–5.45, *p* < 0.001) and collective action intentions (*t*_174_–4.14, *p* < 0.001). CSI participants and non-participants’ intergroup attitudes did not differ significantly from one another, however (*t*_159_–0.1, *p* = 0.46). There were significant differences between host and displaced participants’ (Host = 0, Displaced = 1) cross-group friendship (*t*_169_–3.58, *p* < 0.001) and intergroup attitudes (*t*_159_ 7.04, *p* < 0.001). Host and displaced participants did not significantly differ in collective action intentions, however (*t*_174_–0.36, *p* = 0.35). Finally, we conducted bivariate correlations to check for multicollinearity among our continuous dependent variables (cross-group friendship, collective action intentions, and intergroup attitudes). None of the variables were highly correlated, indicating that multicollinearity was not present (See Table 4).

**Table 4:** Means, standard deviations and correlations.

	Variable	M	SD	1	2
1	Attitudes	75.6	20.687		
2	Collective action	4.67	1.263	0.129	
3	Cross-group friendship	3.07	1.975	0.279^**^	0.224^**^

^**^Correlation is significant at the 0.01 level (two-tailed).

### Effect of CSI participation on intergroup outcomes

We conducted three 2 (CSI: Yes vs. No) × 2 (Resident/nationals vs. displaced persons) between-subjects ANOVAs to test the effects of CSI participation for residents/nationals and displaced persons on cross-group friendship, collective action, and outgroup attitudes (See Table 5).

**Table 5:** 2 × 2 ANOVA: Descriptive statistics and results.

Variable	CSI: Yes		CSI: No		ANOVA			
	*M*	SD	*M*	SD	Effect	F	df	*p*
Friendship
Host	3.59	2.1	1.52	1.04	CSI	16.6	1,167	<0.001
DP	3.65	1.82	3.22	2.15	G	8.26	1,167	0.005
					CSI * G	7.12	1,167	0.008
Collective action
Host	5.06	0.87	4.23	1.11	CSI	17.44	1,172	<0.001
DP	4.90	1.31	4.09	1.52	G	0.565	1,172	0.45
					CSI* G	0.00	1,172	0.984
Attitudes
Host	90.39	9.0	81.63	17.6	CSI	3.6	1,157	0.06
DP	66.45	21.25	63.07	20.07	G	48.1	1,157	<0.001
					CSI* G	1.01	1,157	0.31

* G = group (Host/DP).

#### Cross-group friendship

**Figure 2:**
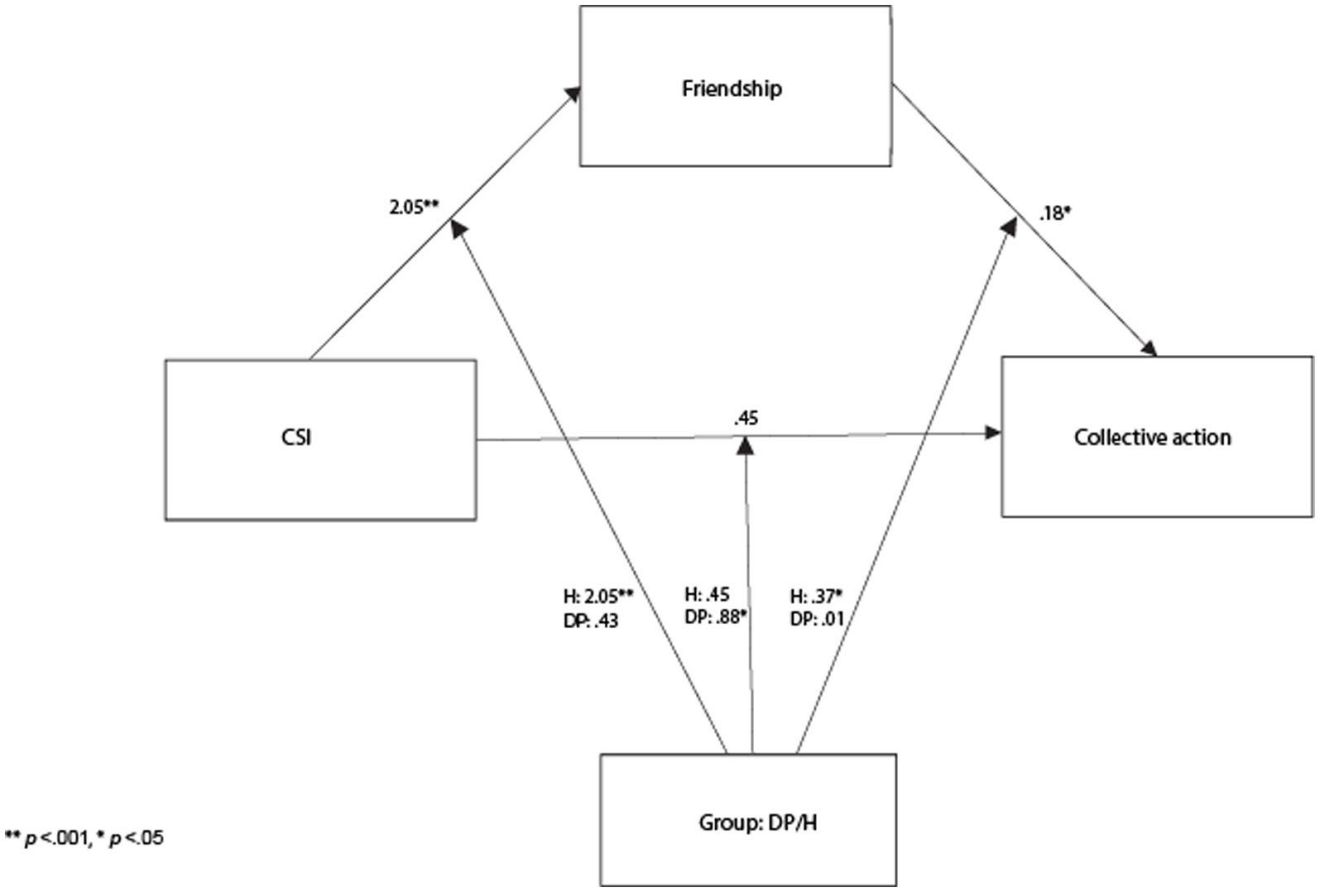
Moderated mediation model.

In line with our predictions, the main effect of CSI was significant (*F*_1,167_ = 16.6, *p*<0.001), such that participants who had engaged with CSI reported more contact with cross-group friends (*M* = 3.63, *SD* = 1.91) than those who did not (*M* = 2.03, *SD* = 1.65). The main effect for Group was also significant (*F*_1,167_ = 8.26_,_ *p* = 0.005), and resident/nationals reported less contact with cross-group friends (*M* = 2.52, *SD* = 1.93) than displaced persons (*M* = 3.57, *SD* = 1.89). The interaction effect of CSI and Group was also significant (*F*_1,167_ = 7.12, *p* = 0.008). Participation in CSI had a stronger effect on resident/nationals’ contact with cross-group friends, and residents/national participants of CSI reported slightly more contact with cross-group friends than displaced CSI participants (See Figure 2). In other words, participation in CSI was associated with more contact with cross-group friends for both resident/nationals and displaced participants, but CSI participation was related to bigger increases in cross-group friendship for resident/nationals.

#### Collective action

As expected, the main effect for CSI participation was significant (*F*_1,172_ = 17.44, *p*<0.001), meaning that participants of CSI reported slightly stronger collective action intentions (*M* = 4.96, *SD* = 1.17) than those who did not (*M* = 4.18, *SD* = 1.27). Contrary to our expectations, however, neither the main effect for group (*F*_1,172_ = 0.57, *p* = 0.45) nor the interaction (*F*_1,172_ = 0.00 *p* = 0.98) was significant. In other words, CSI participants were more likely to support taking collective action in support of displaced people’s rights than people who had never taken part in a CSI, regardless of group membership. Importantly, there were no significant differences between collective action intentions of displaced participants and non-participants of CSI.

#### Outgroup attitudes

Contrary to our expectations, the main effect for CSI participation was not significant (*F*_1,157_ = 3.6, *p* = 0.06), meaning that participation in CSI did not seem to influence outgroup attitudes for either group. Furthermore, the main effect for group was significant (*F*_1, 157_ = 48.1, *p* < 0.001), meaning that resident/national participants expressed more positive outgroup attitudes (*M* = 85.85, *SD* = 14.75) than displaced people (*M* = 65.73, *SD* = 20.85). The CSI × Group interaction was not significant (*F*_1,157_ = 1.01, *p* = 0.31).

### Conditional process analysis

We employed conditional process analysis to identify whether group membership moderated the effect of CSI participation on collective action intentions through cross-group friendship. To do this, we used the PROCESS macro on SPSS to conduct a moderated mediation, using model 59, specifying *X* as “CSI participation,” *Y* as “collective action intentions,” *M* as “cross-group friendship” and *W* as “group” (coded H/host = 0, DP/displaced = 1) for all pathways. We report the unstandardized effect sizes following guidance of Hayes (2018) for dichotomous moderators. The index of moderated mediation was significant: (*B* = −0.3, *SE* = 0.15, LLCI: −0.76; ULCI: −0.15), group membership was a significant moderator for all pathways of the model (Interaction effect *B* = −0.79, *SE* = 0.23, *t* = −3.49, *p* < 0.001, LLCI: −1.24; ULCI.34), and cross-group friendship was positively related to collective action (*B* = 0.48, *SE* = 0.11, *p* = 0.001). For the host group, participation in CSI was positively associated with collective action intentions through increased levels of contact with cross-group friends (Effect = 0.37, *SE* = 0.13, LLCI: 0.1247, ULCI: 0.65). However, for the displaced group participation in CSI was not related to collective action intentions through cross-group friendship (Effect: 0.01, *SE* = 0.15, LLCI: −0.1064; ULCI: 0.1604; See Figure 2).

## Discussion

We investigated whether and how participation in CSI was associated with cross-group friendships, collective action intentions, and intergroup attitudes for displaced people and resident/nationals, and we found that CSI participation was positively associated with cross-group friendship and collective action intentions in support of displaced people’s rights for both displaced people and resident/nationals. For resident/nationals, participation in the CSI was associated with stronger collective action intentions through increased contact with cross-group friends; however, this relationship was not observed for displaced CSI participants. Although participation in a CSI did not predict more positive intergroup attitudes, we observed that displaced people had more ambivalent intergroup attitudes than resident/nationals. Taken together, these findings provide insights to the different perspectives of displaced and resident/nationals participants of CSI, highlighting the importance of CSI in mobilizing collective action intentions in support of displaced people’s rights, and the key role of cross-group friendship in sustaining collective action intentions for resident/nationals.

CSI participants from both groups reported more contact with cross-group friends, indicating that CSI facilitate positive intergroup contact, where meaningful cross-group relationships can be developed. CSI participation was also related to stronger collective action intentions for both groups, suggesting that people who engage in CSI express stronger intentions to take collective action in support of displaced people’s rights. Because CSI are founded on principles of solidarity with displaced people, resident/national participants are likely to be welcoming toward displaced people, although they may vary in their political backgrounds or preferred social change strategies (Kende et al., 2017; Vine and Greenwood, 2022). Therefore, displaced people may see CSI as safer spaces where they can meet and make friends with resident/nationals. Nevertheless, contact in CSI occurs within a broader context of entrenched inequality, and both groups experience intergroup contact differently as a result. For example, our previous research identified that residents/nationals navigated dilemmas related to their power and privilege in CSI, and displaced people negotiated their identities to contest negative social representations of their group (Vine and Greenwood 2020, 2022). Therefore, although shared activities may *temporarily* break down boundaries between groups, the power asymmetries between displaced people and resident/nationals still shape people’s experiences of CSI, and any effects of positive contact are likely to be limited as a result (Mousa, 2020).

Our findings suggest that CSI offer resident/nationals and displaced people opportunities to forge bonds of friendship that can sustain commitment to social change and contribute to building social cohesion. We investigated the relationship between CSI participation and collective action further and found that it looks different for resident/nationals and displaced people. For resident/national participants of CSI, increased contact with displaced friends predicted stronger collective action intentions. Cross-group friendships facilitate emotional connection, and processes of *decategorization* that make shared “human” identities more salient (Brewer and Miller, 1984; Brown et al., 1999; Gaertner et al., 1999). Accordingly, cross-group friendships may afford resident/nationals opportunities to learn what it is like to live in DP centers and gain insight into the structural injustice residents endure, which may fuel resident/nationals’ collective action intentions. Thus, CSI may function as the soil in which cross-group friendships can take root and nurture resident/nationals’ political solidarity with displaced people. In essence, CSI serve as important mediating structures through which residents/nationals can align in political solidarity with displaced people to contest the illegitimacy of the DP system and work together for social change (Subašić et al., 2008; Neufeld et al., 2019; Greenwood, 2008). These findings are in line with previous findings that even host volunteers who engaged with “apolitical” solidarity initiatives were motivated by desires for social change (Fleischmann and Steinhilper, 2017; Kende et al., 2017). In this way, CSI participation may indirectly support social cohesion through strengthening of intergroup bonds and motivating action for a common purpose.

Group membership was a key factor in the relationship between cross-group friendship and collective action. The content of the contact in CSI may partially explain this finding: if cross-group friendships for CSI participants focused on similarities more than differences, and if resident/nationals did not openly discuss the illegitimacy of intergroup inequality, then cross-group friendships may not motivate collective action for displaced people (MacInnis and Hodson, 2019; Hässler et al., 2020b; Tropp et al., 2021). Displaced people and resident/nationals may engage in intergroup contact for different reasons. For example, resident/nationals may seek to feel accepted by displaced people, while displaced people may seek to feel empowered through contact with resident/nationals (Radke et al., 2020; Selvanathan et al., 2020; Hässler et al., 2020b). Hässler et al. (2020b) identified these identity-based needs as important moderators of the relationship between contact and collective action. Cross-group friendships are also likely to serve different functions for displaced people. For example, displaced people may gain access to local resources through supportive friendships with resident/nationals and receive social support while navigating challenges of adapting to a new culture. Although the CSI included in our study are not overtly political, it is possible that engagement with them is a type of informal collective action for displaced people, for whom most avenues to formal collective action are not open (Bekaj and Antara, 2018). Further research is needed to establish whether and how cross-group friendship relates to collective action intentions for displaced people in CSI.

Displaced people reported more ambivalent intergroup attitudes compared to the more positive attitudes of resident/nationals. Disadvantaged group members tend to have more negative experiences of intergroup contact than advantaged group members because of expectations of discrimination and pressure to contest negative stereotypes (Hopkins et al., 2007). Displaced people are predominantly people of the global majority and, as such, are exposed to institutional and direct racism in Irish society (Breen, 2008; Stapleton, 2012; Irish Refugee Council, 2020). Furthermore, the category *asylum seeker* is associated with negative racialised stereotypes and social exclusion. In this broader context of negative contact experiences, it is understandable that displaced people would have relatively ambivalent and guarded attitudes toward cross-group interactions with Irish people as a group (Stephan et al., 2002; Vine and Greenwood, 2020). Our findings are also in line with contact research in contexts of entrenched inequality that identify the limitations of the “contact effect” (Maoz, 2011; Mousa, 2020). Nonetheless, although CSI participation did not have a significant effect on intergroup attitudes, there was a marginally non-significant effect, indicating a trend toward more positive attitudes. Future research with a larger sample could further investigate the relationship between CSI participation and attitudes.

### Limitations and future directions

The present study has some limitations that should be considered. Our participant groups were naturally occurring and not randomly allocated, which prevents us from making causal inferences about the patterns we observed. Furthermore, despite all our efforts to recruit a larger sample, our sample size fell short of our target number, which increased the chances of both false positives and negatives (Button et al., 2013). Participants for this study did indeed prove hard to reach, and we exhausted all avenues that were available to us at the time of data collection. Further data collection for the present study is not feasible, but future research could combine qualitative, participatory, and quantitative approaches to better reflect the community-based nature of the research topic.

Our findings about the role of cross-group friendship in collective action intentions may be partially explained by differences in resident/nationals and displaced peoples’ social change motivations for participating in CSI. People from both groups who take part in CSI may share strong pre-existing collective action intentions, and indeed, some may view engaging in the CSI as a form of collective action. Although this possibility cannot be ruled out, we attempted to address it by excluding participants engaged with overtly political CSI from the analyses (see Table 2).

We used a single item to measure cross-group friendship that assessed *amount of contact with cross-group friends*, taken from Islam and Hewstone’s (1993) contact quantity scale and adapted for our study. Single-item measures are often used for brevity in survey research, but they often have low construct validity. Our measure assessed only quantity, and not quality or closeness. However, a large meta-analysis on cross-group friendships and intergroup attitudes identified that studies using single-item measures, and measures of contact quantity still yielded moderate effect sizes (Davies et al., 2011). Future research should investigate the importance of other features of cross-group friendships, such as trust, to collective action.

### Conclusion

As the global rate of displacement rises, alongside an ever-tightening and violent border-industrial complex, solidarity among displaced persons and resident/nationals is increasingly urgent. Our research highlights the important relationship between participation in CSI, cross-group friendship, and political solidarity among displaced people and resident/nationals. By creating conditions for intergroup solidarity to flourish, CSI may indirectly sustain movements for social change in favor of displaced people’s rights, bolstering social cohesion. Our research demonstrates the benefits of CSI based on collaborative contact that encourages meaningful cross-group friendships, over transactional types of contact (e.g., giving donations). These findings are relevant for practitioners, community members, and organizations that work alongside displaced people in their receiving countries.

## Data availability statement

The raw data supporting the conclusions of this article will be made available by the authors, without undue reservation.

## Ethics statement

The studies involving human participants were reviewed and approved by UL Education and Health Sciences Research Ethics Committee. Reference number: 2020_05_08. The patients/participants provided their written informed consent to participate in this study.

## Author contributions

MV designed the study, collected the data, analyzed the data, and wrote the manuscript. RG supervised and advised the MV throughout the research project, and edited the manuscript.

## Funding

This research was funded by the Irish Research Council Postgraduate scholarship, GOIPG/2019/2474.

## Acknowledgments

We would like to acknowledge the contributions of Aimen Kakar, Owudunni Ola Mustapha, Andile Mondela, Jihad Essektani, Vulez Akinde, Hala Jaber, and Redha Abbood who supported this research project in the design and data collection phases. We would also like to express our appreciation to reviewers Ozge Savas and Seth Green for their insights and suggestions which helped to improve this manuscript.

## Conflict of interest

The authors declare that the research was conducted in the absence of any commercial or financial relationships that could be construed as a potential conflict of interest.

The reviewer ÖS declared a past co-authorship with the author RG to the handling editor.

## Publisher’s note

All claims expressed in this article are solely those of the authors and do not necessarily represent those of their affiliated organizations, or those of the publisher, the editors and the reviewers. Any product that may be evaluated in this article, or claim that may be made by its manufacturer, is not guaranteed or endorsed by the publisher.

## Supplementary material

The Supplementary material for this article can be found online at: https://www.frontiersin.org/articles/10.3389/fpsyg.2023.1042577/full#supplementary-material

## Footnotes

^1^We use the term "displaced people" in this paper to refer to people living within Direct Provision (DP). Most people in DP are waiting for their applications for international protection to be processed. In Ireland, asylum seekers can apply for refugee status, subsidiary protection or humanitarian leave to remain. For more information on the Irish asylum system please see the AIDA website: https://asylumineurope.org/reports/country/republic-ireland/
