# Supplementary material for: Cross-group friendship and collective action in community solidarity initiatives with displaced people and resident/nationals
Source: Front Psychol. 2023 Apr 3;14:1042577. doi: 10.3389/fpsyg.2023.1042577 (PMC10106771; doi:10.3389/fpsyg.2023.1042577)
Supplement: Supplementary file 1 [file Data_Sheet_1.PDF]

Experiences of community solidarity initiatives: Information Sheet

What is the project about? In this study we want to understand how people's experiences of contact with members of other groups can influence how they adapt to other cultures, their feelings about other groups, and their well-being. Community solidarity initiatives that bring together displaced people (asylum seekers and refugees) and the host community are an example of a situation where members of different groups come into contact in Ireland. In our study, we are interested in the experiences of displaced people and the host community who have taken part in community solidarity initiatives in the past year. We would also like to hear from those groups of people who have not taken part in these initiatives to understand the effects of intergroup contact more generally.

What will I have to do? If you agree to be part of this study, you will be asked to complete a survey about your experiences of contact with members of other groups, your engagement in different cultural activities, your feelings toward members of other groups, and your well-being. We recommend that you do the survey when you have some time to focus, using a pen or pencil. When you have completed the survey, please seal the survey in the numbered envelope, and give it back to the person in your centre who was distributing the surveys. That person will then post the surveys to the research team.

What are the benefits? Taking part in this survey may give you insight into your thoughts and feelings, which may be beneficial to you.

What are the risks? Some of the questions in this research study ask about your psychological well-being, which may be difficult for some people. You can skip over any questions that make you feel uncomfortable, and if you do begin to feel uncomfortable answering these questions, you can stop participating at any time.

What if I do not want to take part? Your participation in this study is completely voluntary. You do not have to take part if you do not want to, and you can stop participating at any time. Whether you participate or not will not be known to the researchers or anyone else. Any supports, services, or resources you receive will not be affected by whether you participate or not. Whether you participate cannot affect your asylum applications or residence status.

What happens to the information? All data will be maintained as confidential. We will ask you for some personal data such as your age, nationality, and gender. The data will be stored on the researcher's password-protected computer for 7 years, after which they will be deleted. Any paper documents back-up

files of data stored on flash drives, or external hard drives will be password protected securely kept in a locked filing cabinet in the researcher's desk for 7 years and will then be destroyed.

Who else is taking part? Displaced people (people who are asylum seekers and refugees) as well as members of the host community of Ireland are invited to take part in this research. All participants must be currently living in Ireland and must be over 18 years of age to take part.

What happens at the end of the study? At the end of the study, the results will be analysed and written as part of Megan Vine's doctoral dissertation. They will also be written in the form of a manuscript for publication. A summary of the findings will be made available to all participants, on request.

What if I change my mind during the study? You may withdraw from the study at any point, for any reason, if you do not wish to continue with participation.

What if I have more questions or do not understand something? If you have any questions at any time, please feel free to contact a member of the research team. Our contact information is below:

Researcher Contact Information:

Principal Investigator: Megan Vine, PhD student, Psychology Department, Tel (085) 8226449.
Email: megan.vine@ul.ie.

Other investigators: Dr Ronni Greenwood, Department of Psychology. University of Limerick, Tel (061) 234618. Email: ronni.greenwood@ul.ie

This research study has received ethical approval from the Education and Health Sciences Research Ethics Committee [2020_05_08].

If you have any concerns about this study and wish to contact someone independent, you may contact the Chairperson of the Education and Health Sciences Research Ethics Committee, EHS Faculty Office, University of Limerick, Tel (061)234101.
Email: ehsresearchethics@ul.ie

Participant Consent

Please read the statements below and if you agree to them, please select 'Yes' below. If you do not agree, select 'No'.

- I have read and understood the participant information.
- I understand what the project is about, and what the results will be used for.
- I understand that what the researchers find out in this study may be shared with others, but that I will not be identified in any way.
- I am fully aware of what I will have to do, and of any risks and benefits of the study.
- I know that I am choosing to take part in the study and that I can stop taking part in the study at any stage without giving any reason to the researchers.

Do you consent to participate in this study? Please select the appropriate answer below*

○ Yes

○ No

If you give consent to participate, please sign below:

X ____________________

Research Privacy Notice

This Privacy Notice governs the use and storage of your personal data by the University of Limerick (the University). The processing of this data is carried out in accordance with the General Data Protection Regulation (GDPR) / Data Protection Acts 1988-2018 (“Data Protection Law”) and in accordance with this Data Protection Privacy Notice. The University is the Data Controller for personal data we process about you. The purpose of this Data Protection Privacy Notice is to explain how the University uses and processes personal data we collect and hold about you as a research participant (“you”, “your”). This notice extends to all your personal data as defined under Article 2(1) of the General Data Protection Regulation (EU) 2016/679.

1. Title and Purpose of the research project

1.1 This study is titled, ‘Experiences of community solidarity initiatives’. The purpose of the research is primarily to ascertain whether, and how, community solidarity initiatives (CSI) achieve their aims of improving relations between asylum seekers and refugees (displaced people) and the wider Irish community (the host community), and to identify the psycho-social impacts of participation for host and displaced participants.

1.2 Potential benefits that may arise from the research project
Participating in this survey may benefit people in terms of gaining a personal insight into their behaviours, attitudes and well-being. Furthermore, participants will gain an insight into how research is done, which may be beneficial for those who are interested in pursuing a career in academia or research.

2. Research Ethics Committee

2.1 Ethical approval was granted by the EHS Research Ethics Committee on 15/5/2020.

3. Identity of the Data Controller(s)

3.1 The Data Controller is University of Limerick, Plassey, Limerick.
4.1 You can contact the University of Limerick’s Data Protection Officer at dataprotection@ul.ie or by writing to Data Protection Officer, Room A1-073, University of Limerick, Plassey, Limerick.

5. The Identity of the Principal Investigator

5.1The Principal Investigator for this Research Project is Megan Vine, PhD researcher at the University of Limerick.

6. Why the University Holds Your Personal Data

6.1 The University must process your personal data in order to undertake research relating to people’s experiences of community solidarity initiatives.

7. Research Participant Personal Data held by the University

7.1 You provide us with your personal data to enable us to undertake the research project. Participation in this research project is voluntary and participants may withdraw without giving any reason. Should you wish to withdraw you may do so by contacting the Principal Investigator at megan.vine@ul.ie or writing to her at Psychology Department, University of Limerick, County Limerick.
7.2 The categories of personal data collected/recorded may include:

nationality, racial/ethnic origin, gender, age, region, residence status.

8. Lawful Basis for University Processing Personal Data

8.1 Data Protection Law requires that the University must have a valid lawful basis in order to process personal data.
8.2 The University will rely on your explicit consent in order to process your personal data for research purposes. Consent must be freely given, specific, informed and an unambiguous indication of your wishes by which you (by a statement or by a clear affirmative action) signify agreement to the processing of personal data relating to you. Your decision not to consent will have no adverse consequences for you.
8.3 You are free to withdraw this consent and you can do so by contacting the Principal Investigator at the following email address: megan.vine@ul.ie.

9. Protecting Your Personal Data

9.1 Reasonable appropriate administrative, technical, personnel procedural and physical measures are employed to safeguard Personal Data against loss, theft and unauthorised uses access, uses or modifications.
9.2 All researchers of the University must adhere to the University's Data Protection Policy when processing Personal Data on behalf of UL (available at www.ul.ie/dataprotection). Non-adherence to the University's Data Protection Policy may lead to disciplinary action. Researchers of the University shall undertake Data Protection Training before they engage in the research project.
9.3 Personal data collected for this research project will be pseudonymised within 1 month after collection and will fully anonymised within/after 12 months. Truly anonymised data is not Personal Data. Once data is anonymised for the purposes of this research project, the terms of this Privacy Notice will no longer apply.

10. Sharing Your Personal Data with Third Parties

10.1 The University will not disclose your personal data to third parties.

11. Retention of your Personal Data

11.1 All Personal Data collected for this research project will be retained in accordance with the University's Records Management and Retention Policy.

12. Your Rights

12.1 Depending on the legal basis which we rely on to process your Personal Data, you may have the right to request that we:

- provide you with information as to whether we process your data and details relating to our processing, and with a copy of your personal data;
- rectify any inaccurate data we might have about you without undue delay;
- complete any incomplete information about you;
- under certain circumstances, erase your Personal Data without undue delay;
- under certain circumstances, be restricted from processing your data;
- under certain circumstances, furnish you with the Personal Data which you provided us within a structured, commonly used and machine readable format;

12.2 Requests for any of the above should be addressed by email to the Principal Investigator at megan.vine@ul.ie AND the Data Protection Officer at dataprotection@ul.ie. Your request will be processed within 30 days of receipt. Please note, however, it may not be possible to facilitate all requests, for example, where the University is required by law to collect and process certain personal data including that personal information that is required of any research participant.

12.3 It is your responsibility to let the Principal Investigator know if your contact details change.

13. Queries, Contacts, Right of Complaint

13.1 Further information on Data Protection at the University of Limerick may be viewed at www.ul.ie/dataprotection. You can contact the Data Protection Officer at dataprotection@ul.ie or by writing to Data Protection Officer, Room A1-073, University of Limerick, Limerick.

13.2 You have a right to lodge a complaint with the Office of the Data Protection Commissioner (Supervisory Authority). While we recommend that you raise any concerns or queries with us first at the following email address megan.vine@ul.ie, you may contact that Office at info@dataprotection.ie or by writing to the Data Protection Commissioner, Canal House, Station Road, Portarlington, Co. Laois.

14. Review

14.1 This Privacy Notice will be reviewed and updated from time to time to take into account changes in the law and the experience gained from the Notice in practice.

Do you agree with the terms of this Research Privacy Notice?

○ Yes

○ No

If you agree, please sign below:

X ____________________

Experiences of community solidarity initiatives: Survey

Please select which sentence describes you: *

- ○ I am currently living in Ireland, and I am currently in the process of seeking international protection OR I have applied for international protection in Ireland within the past 5 years
- ○ I have been resident in Ireland for 5 or more years, and I have never sought asylum in Ireland

*Answer required

In the past year, have you taken part in any organised activity as part of a community group that brings together displaced people (asylum seekers and refugees) and members of the host community? This might be a sports club, a cooking group, a cultural celebration, or any other community activity that aims to bring people of different backgrounds into contact with one another.
Note: In the rest of this survey we will call these groups 'community solidarity initiatives'. *

- ○ Yes
- ○ No

*Answer required

If you answered yes to the above, please write the name(s) of the initiative(s) that you have taken part in below:

____

____

What is your nationality? (Please write below)

____

Please show how much you disagree or agree with each the four statements below. For these questions please think about your national group (people who have the same nationality as you).

	Strongly Disagree	Slightly disagree	Somewhat disagree	Neither agree nor disagree	Somewhat agree	Agree	Strongly agree
1.I feel a bond with my national group	○	○	○	○	○	○	○
2.I feel similar to the other members of my national group	○	○	○	○	○	○	○
3.I have a sense of belonging to my national group	○	○	○	○	○	○	○
4.I have a lot in common with the members of my national group	○	○	○	○	○	○	○

Where in Ireland do you live? Please write the county and area below.
County ____
Area ____

Please show how much you disagree or agree with each the four statements below. For these questions, please think about the community of people near where you live.

	Strongly disagree	Disagree	Somewhat disagree	Neither agree nor disagree	Somewhat agree	Agree	Strongly agree
1.I feel a bond with my local community	○	○	○	○	○	○	○
2.I feel similar to the other members of my local community	○	○	○	○	○	○	○
3.I have a sense of belonging to my local community	○	○	○	○	○	○	○
4.I have a lot in common with the members of my local community	○	○	○	○	○	○	○

Please indicate your level of agreement with each of the following statements by selecting a response on the scale below. When answering the following questions, please think about people who are members of the host community of Ireland.

	No knowledge	Very little knowledge	Some knowledge	A moderate amount of knowledge	A fair amount of knowledge	A good amount of knowledge	A lot of knowledge
1. In general, how much do you know about the host community?	○	○	○	○	○	○	○
2. In general, how much do you know about the host community's history?	○	○	○	○	○	○	○
3. In general, how much do you know about the host community's languages?	○	○	○	○	○	○	○
4. In general, how much do you know about the host community's values?	○	○	○	○	○	○	○

Please indicate your level of agreement with the following statement by selecting a response on the scale below. When answering the following question, please think about people who are members of the host community of Ireland.

	Definitely take advantage	Maybe take advantage	Not sure	Maybe try to be fair	Definitely try to be fair
Do you think most people in the host community would try to take advantage of you if they got a chance, or would they try to be fair?	○	○	○	○	○

Please indicate your level of agreement with the following statement by selecting a response on the scale below. When answering the following question, please think about people who are members of the host community of Ireland.

	Definitely try to be helpful	Maybe try to be helpful	Not sure	Maybe out for themselves	Definitely out for themselves
Would you say that most of the time people in the host community try to be helpful, or that they are mostly just looking out for themselves?	○	○	○	○	○

UNIVERSITY OF LIMERICK
OLLSCOIL LUIMNIGH

Please indicate your level of agreement with the following statement by selecting a response on the scale below. When answering the following question, please think about people who are members of the host community of Ireland.

	Definitely can be trusted	Maybe can be trusted	Not sure	Maybe can't be too careful	Definitely can't be too careful
Generally speaking, would you say that most people in the host community can be trusted or that you can't be too careful with them?	○	○	○	○	○

Please indicate your level of agreement with each of the following statements by selecting a response on the scale shown below.

	Strongly Disagree	Disagree	Somewhat disagree	Not sure	Somewhat agree	Agree	Strongly Agree
1. Usually, when I deal with people in the host community, I feel tense	○	○	○	○	○	○	○
2. Usually, when I deal with people in the host community, I feel threatened	○	○	○	○	○	○	○
3. Usually, when I deal with people in the host community, I feel uncomfortable	○	○	○	○	○	○	○
4.Usually, when I deal with people in the host community, I feel nervous	○	○	○	○	○	○	○

Please indicate your answer for each of the following questions by selecting a number on the scales shown below. How much contact do you have with members of the host community?

	None at all	Very little	A little	Some contact	A fair amount	A lot	A large amount
at college/work	○	○	○	○	○	○	○
as neighbours	○	○	○	○	○	○	○
as close friends	○	○	○	○	○	○	○

Please indicate your answer for each of the following questions by selecting a number on the scales shown below. How often have you:

	Never	Once	A couple of times	A few times	Monthly	Weekly	Daily
engaged in informal conversations with people in the host community	○	○	○	○	○	○	○
visited the homes of people in the host community	○	○	○	○	○	○	○

Please indicate your answer for each of the following questions by selecting a response on the scale shown below.

	Not at all equal	Moderately unequal	A little unequal	Neutral	A little equal	Moderately equal	Very equal
To what extent did you experience the contact with members of the host community as equal?	○	○	○	○	○	○	○

Please indicate your answer for each of the following questions by selecting a response on the scale shown below.

	Definitely voluntary	Moderately voluntary	Somewhat voluntary	Neutral	Somewhat involuntary	Moderately involuntary	Definitely involuntary
To what extent did you experience the contact with the host community as involuntary or voluntary?	○	○	○	○	○	○	○

Please indicate your answer for each of the following questions by selecting a response on the scale shown below.

	Very superficial	Moderately superficial	Somewhat superficial	Neutral	Somewhat intimate	Moderately intimate	Very intimate
To what extent did you experience the contact with members of the host community as superficial or intimate?	○	○	○	○	○	○	○

Please indicate your answer for each of the following questions by selecting a response on the scale shown below.

	Not at all pleasant	Moderately unpleasant	Somewhat unpleasant	Neutral	Somewhat pleasant	Moderately pleasant	Very pleasant
To what extent did you experience the contact with members of the host community as pleasant?	○	○	○	○	○	○	○

Please indicate your answer for each of the following questions by selecting a response on the scale shown below.

	Very competitive	Moderately competitive	Somewhat competitive	Neutral	Somewhat cooperative	Moderately cooperative	Very cooperative
To what extent did you experience the contact with members of the host community as competitive or cooperative?	○	○	○	○	○	○	○

Below is something that looks like a thermometer. We call it a 'feeling thermometer' because it measures your feelings towards groups. Here's how it works. If you don't know too much about a group or don't feel particularly warm or cold about them, then you should place them in the middle, at the 50 degree mark. If you have a warm feeling towards a group or feel favourably toward it, you would give it a score somewhere between 50 and 100 depending on how warm your feeling is toward the group. On the other hand if you don't feel very favourably toward some of these groups, if there are some you don't care for too much then you would place them somewhere between the 0 and 50 mark. When answering the following question, please think about people who are members of the host community of Ireland.

Feelings towards the host community in Ireland

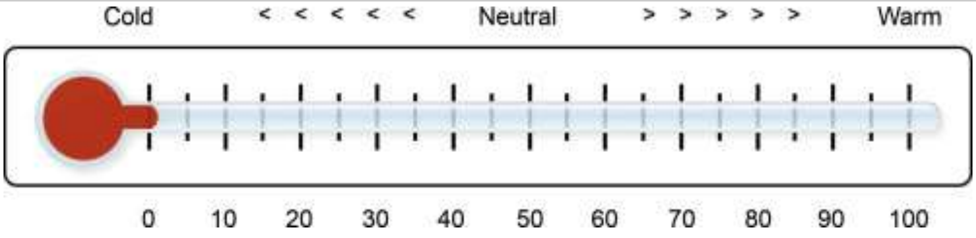

Please think about collective actions you might get involved with in the future to support the rights of other asylum seekers and refugees when answering the following questions. Please select a response for each of the following statements to show how often you intend to take part in these actions in the future.

	Never	A little	Sometimes	Quite a bit	Often	Very often
1. I will support political candidates who made the rights of displaced people, e.g. ending Direct Provision, one of their serious concerns.	○	○	○	○	○	○
2. I will participate in demonstrations that call for the recognition of the rights of displaced people.	○	○	○	○	○	○
3. I will sign a petition against displaced people being disadvantaged compared to citizens.	○	○	○	○	○	○
4. I will join a group of activists demanding the recognition of the rights of displaced people.	○	○	○	○	○	○
5. I will attend events in which people were informed about and discussed issues relating to displaced people.	○	○	○	○	○	○
6. I will defend displaced people’s rights in discussions with peers, colleagues, relatives, and/or friends.	○	○	○	○	○	○
7. I will support an displaced person against discrimination	○	○	○	○	○	○

Many of these questions will refer to your heritage culture, meaning the original culture of your family (other than Irish). It may be the culture of your birth, the culture in which you have been raised, or any culture in your family background. If there are several, pick the one that has influenced you most. Please show your degree of agreement or disagreement with each of the 20 statements on the scale below.

	Strongly Disagree	Disagree	Somewhat disagree	Neither agree nor disagree	Somewhat agree	Agree	Strongly agree
1. I often participate in my heritage cultural traditions.	○	○	○	○	○	○	○
2. I often participate in mainstream Irish cultural traditions.	○	○	○	○	○	○	○
3. I would be willing to marry a person from my heritage culture.	○	○	○	○	○	○	○
4. I would be willing to marry an Irish person.	○	○	○	○	○	○	○
5. I enjoy social activities with people from the same heritage culture as myself	○	○	○	○	○	○	○
6. I enjoy social activities with typical Irish people.	○	○	○	○	○	○	○
7. I am comfortable interacting with people of the same heritage culture as myself.	○	○	○	○	○	○	○

8. I am comfortable interacting with typical Irish people.	○	○	○	○	○	○	○
9. I enjoy entertainment (e.g. movies, music) from my heritage culture.	○	○	○	○	○	○	○
10. I enjoy Irish entertainment (e.g. movies, music).	○	○	○	○	○	○	○
11. I often behave in ways that are typical of my heritage culture.	○	○	○	○	○	○	○
12. I often behave in ways that are typically Irish.	○	○	○	○	○	○	○
13. It is important for me to maintain or develop the practices of my heritage culture.	○	○	○	○	○	○	○
14. It is important for me to maintain or develop Irish cultural practices..	○	○	○	○	○	○	○
15. I believe in the values of my heritage culture.	○	○	○	○	○	○	○

16. I believe in mainstream Irish values.	○	○	○	○	○	○	○
17. I enjoy the jokes and humour of my heritage culture.	○	○	○	○	○	○	○
18. I enjoy Irish jokes and humour.	○	○	○	○	○	○	○
19. I am interested in having friends from my heritage culture.	○	○	○	○	○	○	○
20. I am interested in having Irish friends.	○	○	○	○	○	○	○

The following 18 questions are about your psychological well-being.
Please select a response on the scale below for each statement to indicate how much you agree or disagree.

	Strongly disagree	Disagree	Somewhat disagree	Neither agree nor disagree	Somewhat agree	Agree	Strongly agree
1. I like most parts of my personality	○	○	○	○	○	○	○
2. When I look at the story of my life, I am pleased with how things have turned out so far	○	○	○	○	○	○	○
3. Some people wander aimlessly through life, but I am not one of them	○	○	○	○	○	○	○
4. The demands of everyday life often get me down	○	○	○	○	○	○	○
5. In many ways I feel disappointed about my achievements in life	○	○	○	○	○	○	○
6. Maintaining close relationships has been difficult and frustrating for me	○	○	○	○	○	○	○
7. I live life one day at a time and don't really think about the future	○	○	○	○	○	○	○
8. In general, I feel I am in charge of the situation in which I live	○	○	○	○	○	○	○
9. I am good at managing the responsibilities of daily life.	○	○	○	○	○	○	○
10. I sometimes feel as if I've done all there is to do in life.	○	○	○	○	○	○	○
11. For me, life has been a continuous process of learning, changing, and growth	○	○	○	○	○	○	○

UNIVERSITY OF LIMERICK
OLLSCOIL LUIMNIGH

12. I think it is important to have new experiences that challenge how I think about myself and the world.	○	○	○	○	○	○	○
13. People would describe me as a giving person, willing to share my time with others	○	○	○	○	○	○	○
14. I gave up trying to make big improvements or changes in my life a long time ago	○	○	○	○	○	○	○
15. I tend to be influenced by people with strong opinions	○	○	○	○	○	○	○
16. I have not experienced many warm and trusting relationships with others.	○	○	○	○	○	○	○
17. I have confidence in my own opinions, even if they are different from the way most other people think	○	○	○	○	○	○	○
18. I judge myself by what I think is important, not by the values of what others think is important	○	○	○	○	○	○	○

What is your gender?

○ Female

○ Male

○ Other

UNIVERSITY OF LIMERICK
OLLSCOIL LUIMNIGH

What is your age?

- ◯ 18-26
- ◯ 27 -38
- ◯ 40+
- ◯ 50+
- ◯ 60+
- ◯ 70+

What is your ethnicity?

- ◯ Irish
- ◯ Irish Traveller
- ◯ Any other White background
- ◯ African
- ◯ Any other Black background
- ◯ Chinese
- ◯ Any other Asian background
- ◯ Other (including mixed background)

What is your immigration status?

- Irish citizen
- EU citizen
- Asylum seeker
- Refugee
- Subsidiary protection
- Non EU citizen with stamp 5
- Non EU citizen with stamp 4
- Non EU citizen with stamp 3
- Non EU citizen with stamp 2
- Non EU citizen with stamp 1

How long have you lived continuously in Ireland?
Please select the most appropriate answer on the scale below.

	Less than one year	1-3 years	4-6 years	7-9 years	10-12 years	13-15 years	16+ years
Time living in Ireland	○	○	○	○	○	○	○

Thank you for participating in our survey, we really appreciate you taking the time to do it.

Would you like to enter a raffle for the chance to win one of 4 50 Euro One4All vouchers?

Please scan the QR code below with your phone to enter your contact details for the raffle.

Please seal your survey in the numbered envelope provided and return it to the person who was distributing the surveys.

(Extra copies of the information sheet have also been provided for you to keep.)
